# Applications for oral research in microgravity – lessons learned from burning mouth syndrome and ageing studies

**DOI:** 10.25122/jml-2022-0285

**Published:** 2023-03

**Authors:** Cosmin Dugan, Ioanina Parlatescu, Bogdan Ovidiu Popescu, Corina Silvia Pop, Mihaela Marin, Adrian Dinculescu, Alexandru Ion Nistorescu, Cristian Vizitiu, Valentin Nicolae Varlas

**Affiliations:** 1 Internal Medicine Department, Bucharest University Emergency Hospital, Bucharest, Romania; 2 Faculty of General Medicine, Carol Davila University of Medicine and Pharmacy, Bucharest, Romania; 3 Faculty of Dentistry, Carol Davila University of Medicine and Pharmacy, Bucharest, Romania; 4 Space Applications for Health and Safety Laboratory, Institute of Space Science, Magurele, Romania; 5 Department of Automatics and Information Technology, Faculty of Electrical Engineering and Computer Science, Transilvania University of Brasov, Brasov, Romania; 6 Department of Obstetrics and Gynaecology, Clinical Hospital of Obstetrics and Gynecology Filantropia, Bucharest, Romania

**Keywords:** microgravity, TMS, tDCS, ageing, oral physiology, burning mouth syndrome

## Abstract

The negative consequences of microgravity for the human body are central aspects of space travel that raise health problems. Altered functions of the same systems and treatment options are common points of spaceflight physiology, age-related diseases, and oral medicine. This work emphasizes the convergence of knowledge between pathophysiological changes brought on by aging, physiological reactions to microgravity exposure, and non-pharmacological and non-invasive treatment methods that can be used in spaceflight. Sarcopenia, peripheral nerves alterations, neuromotor plaque in the masticatory muscles, lingual, labial, and buccal weakness, nociplastic pain in oral mucosal diseases, and microgravity, as well as soft tissue changes and pathologies related to chewing and swallowing, corticomotor neuroplasticity of tongue, and swallowing biomechanics, are of particular interest to us. Neurologic disease and other pathologies such as recovery from post-stroke dysphagia, nociplastic pain in glossodynia, sleep bruxism, and obstructive sleep apnea have been studied and, in some cases, successfully treated with non-invasive direct and transcranial magnetic stimulation (TMS) methods in recent decades. An interdisciplinary team from medical specialties, engineering, and biophysics propose an exploratory study based on the parallelism of ageing and space physiology, along with experiment scenarios considering TMS and non-invasive direct methods.

## INTRODUCTION

Recent research studies have highlighted new correlations between the advancement of space medicine and the physiology of aging [1, 2]. Changes and loss in functional ability are common points in these fields. The effects of microgravity are reported in various human physiological systems, including the musculoskeletal, cardiovascular, cerebral, and sensory-motor systems [1]. Despite astronauts being selected as healthy individuals with reduced risk for diseases, spaceflight can alter their physiology. For example, microgravity conditions can cause muscle atrophy, and some essential components, such as the ability to speak and maintain good oral hygiene, can be compromised. These alterations share similarities with age-related diseases.

Burning mouth syndrome or glossodynia is a chronic oral mucosal disorder characterized by persistent pain without clinical or biological changes. It is a multifactorial disease, and the recent etiology has implicated neuropathic mechanisms. The disease-centered treatment model has been ineffective in providing relief for most patients, so alternative approaches are needed from other medical specialties [3].

Transcranial magnetic stimulation (TMS) and transcranial direct current stimulation (tDCS) are therapeutic methods used to enhance muscular function in the head and neck region, such as recovery from dysphagia post-stroke or improving swallowing function [4]. In addition, TMS is also a treatment option for burning mouth syndrome [5].

This paper presents an exploratory multidisciplinary study for oral physiology and biomechanics research in microgravity and a literature review on microgravity's effects on the head and neck area. We developed a human-in-the-loop muscle assessment procedure that can fit TMS or tDCS therapy for astronauts and the elderly, using parallels between space physiology, aging physiology, and burning mouth syndrome.

## SPACEFLIGHT PHYSIOLOGY AND THE EFFECTS ON THE HEAD AND NECK AREA

During spaceflight, the physiological adaptation to microgravity affects various systems, such as neuro vestibular, cardiovascular, musculoskeletal, bone metabolism, and immunological systems [6]. Rodents are commonly used as animal models in microgravitational research. Studies analyzing the effects of exposure to microgravity on the head and neck region have been conducted on rodents flown on Spacelab-3 (rats), US space shuttles (mice), and the Russian biosatellite Bion-M1 (mice) [7]. Researchers have reported changes in the lower jaw, teeth, and salivary glands. The reduced masticatory activity explains the presence of morphological changes, such as autophagic vacuoles in the acinar cells and more apoptotic cells found in the parotid gland. The expression of salivary proteins varied by flight duration: no changes in parotid glands of mice during the 12.5 days flight on Apollo 17 and a reduced glycoprotein content of submandibular gland saliva of rats flown for 18.5 days on Cosmos 936 and Cosmos 1129 [7].

Loss of body protein and muscle mass and the lack of mechanical forces in microgravity which alters bone resorption and formation were reported in spaceflight animal research studies [8]. Greater bone volume and bone mineral density have been observed in the mouse mandibles during spaceflight compared to vivarium control mice [8]. This was attributable to the altered composition and consistency of the rodent's diet. Another area of the skull that showed a trend to increase in bone volume was the calvaria, both in rodents and astronauts [9]. According to the same authors, space travel can result in unexpected changes in craniofacial bones, determining the risk of dental events in space. Since space missions take place in an isolated environment, it is essential to avoid dental emergencies. However, dental issues have been reported during space travel [9].

The loss of mineral bone density, sleep troubles, and stress due to microgravity affects the temporomandibular joint. During simulated Mars missions, disturbances at the masticatory muscle level have been reported, with increased stiffness observed during space travel [10].

Another part of the oral cavity is the saliva, with various enzymes, electrolytes, and immunologic components. Ivan L et al., in a systematic review, analyzed the oral cavity disturbances both in space conditions and simulated microgravity correlated with spaceflight duration. Long-term space missions reported higher levels of salivary IgA and alteration of the oral microbiome (an increase in the dental plaque's anaerobic components). In short-time missions (10–16 days) and simulated gravity conditions, the increase of metalloproteinases, amylase, and cortisol in saliva was reported [11].

A reduced immunity in the context of stress flight was reported during space missions to the International Space Station and manifested as asymptomatic reactivation of persistent herpes virus infection [12]. In short-term missions, alterations in cell-mediated immunity were detected in astronauts with reactivated herpes virus infections (viral reactivation and shedding of Epstein–Barr virus, varicella-zoster virus, and cytomegalovirus) [13].

The neuroanatomical effects of spaceflight are primarily concerned with the sensorimotor system and the conflict between inputs from visual and tactile senses and vestibular organs. The psychological problems space-connected include a large range of problems linked to anxiety, prolonged isolation, and sleep disturbances [14].

One aging-like effect of microgravity is sarcopenia, which is defined as the involuntary loss of skeletal muscle mass and strength and is associated with age progression [15]. During space missions, exercise programs or special devices for resting are used as countermeasures to prevent sarcopenia [16].

## METHODS AND PRIMARY APPLICATIONS OF TMS AND TDCS

### TMS and tDCS techniques

tDCS is a non-invasive brain stimulation technique that can be used to target specific regions of the brain to treat chronic pain. Positive results have been observed, but more research is needed to determine the efficacy of the therapy as well as the long-term risks involved. Anesthesia, neurorehabilitation, and treatment for depression are a few methods used in medical practice, with considerable documentation supporting their use [17].

Transcranial magnetic stimulation (TMS) uses a changing magnetic field to stimulate a specific cortical region. This is achieved by positioning a coil close to the scalp and generating a low-intensity current to create a changeable magnetic field [18]. The resulting electric field is capable of causing changes in the membrane potentials at the level of the cerebral nerve tissue.

TMS pulses can be used as single pulses (spTMS), pairs of pulses in succession (PP-TMS), or models of recurrent stimulation (1000, 1200, or 1600 stimuli per session). The latter can either be continuous at a certain frequency (rTMS) or programmed with training interstices (*i.e*., intermittent or continuous theta-burst stimulation, iTBS/cTBS) [19].

A magnetic field cannot be focused in the way a lens can focus light, so the method is limited to activating relatively large volumes of tissue compared to a conventional surface electrode. The depth of stimulation is limited by coil design. Currently, advanced mathematical modeling is also used to optimize the degree of precision of TMS stimulations [20].

It is possible to increase the degree to which experiences can be predicted and reproduced, which in turn highlights the variations between individuals when there is enough knowledge about and control over the relevant characteristics. The actual method depends on many characteristics, any of which may change according to the protocol or by mistake. New protocols, such as theta burst stimulation (TBS), have been established and utilized in animal research to elicit synaptic plasticity. These protocols were developed in addition to the traditional repeated stimulation protocols [21].

When attempting to generate an analgesic response via rTMS, one of the essential elements that must be considered is the anatomical placement of stimulation. To this day, the stimulation of anatomical targets for persistent neuropathic pain has been restricted nearly entirely to the motor cortex. The published research includes a diverse range of illnesses, including neuropathic pain, which may manifest in the central or peripheral nervous systems. Trigeminal neuralgia, post-stroke pain (thalamic, lenticular, subcortical, and brainstem-based lesions), and spinal cord damage are all topics investigated in this research [22, 23].

The primary goal of TMS treatment is to alleviate pain in patients. However, pain is a complex phenomenon, and research has identified numerous types of pain, including acute and chronic pain and various subtypes of chronic pain.

The stimulation location is one of the most important factors to consider when trying to elicit an analgesic response after undergoing rTMS. Anatomical stimulation targets for chronic neuropathic pain have primarily been confined almost entirely to the contralateral motor cortex. To the best of our knowledge, only one research [24] attempted to broaden the scope of the stimulation area by applying stimulation to the precentral gyrus, the postcentral gyrus, the premotor region, and the supplementary motor area. Nevertheless, activation of the precentral gyrus was the only factor that resulted in an analgesic response.

In a separate study [25], stimulating the interhemispheric area allowed for simultaneous activation of the lower limb representations on the motor cortex. This was made possible by using a coil in the form of an "H", which is distinct from the almost exclusive use of "8"-shaped coils seen in the other trials. The target pathology is another important factor that distinguishes this study from others in the field. Most are performed on individuals with various etiologies of neuropathic pain, which might lead to considerable disparities in the effects of induced analgesia.

A pilot study involving 12 individuals found that, while six patients reported experiencing significant pain relief, there was no significant difference overall between the placebo and active stimulation when using a circular coil [26]. The remaining repetitive transcranial magnetic stimulation experiments used the figure-8 coil, and the results of these experiments were collected and analyzed in two separate meta-analyses. Both meta-analyses found that low-frequency stimulation did not produce analgesia, while the interpretations of high-frequency stimulation led to varying results.

According to the Cochrane Review [27], which examined single-session stimulation trials using non-invasive brain stimulation techniques, the reduction in pain was only 12%, which was not statistically significant compared to the placebo. There was also a considerable degree of heterogeneity across the studies. Research conducted to study the effects of analgesia over the medium term (less than six weeks) and long term (more than six weeks), which involved following patients for these durations, did not find statistically significant results. Another relevant meta-analysis [28] found that high-frequency repetitive transcranial magnetic stimulation (rTMS) provided analgesia of more than 30% in 46–62% of patients and greater than 50% in 29% of patients. There is also some, if limited, long-term analgesia for the patient's discomfort. It is important to emphasize that the two meta-analyses, which included roughly the same number of trials but selected them differently, did not compare the results with the control group, which likely led to the different interpretations presented in the reviews. As it was previously shown, the unpredictability of the placebo effect in rTMS studies may be connected to the policy of not comparing results to control groups [28].

Even within the same research, the disorders that were evaluated showed substantial diversity. This heterogeneity included both central and peripheral neuropathic pain. This research focuses on the conditions of trigeminal neuralgia, post-stroke pain (originating from thalamic, lenticular, subcortical, and brainstem-based lesions), spinal cord damage, phantom limb pain, nerve root avulsion, and peripheral nerve injury [28].

The meta-analysis by Lefaucheur et al. [29] provides a summary of rTMS studies for complex regional pain syndrome type I (CRPS type I), fibromyalgia, visceral pain, and migraine. The authors found that while some studies showed an analgesic response, the number of studies conducted for these specific pathologies was small, and as a result, no clear conclusions could be drawn. The authors emphasized the need for additional research to fill this knowledge gap.

In order to present a comprehensive view of this treatment development, it is essential to have a conversation about the use of rTMS for conditions other than chronic pain. Studies have been conducted for Parkinson's disease, dystonias, essential tremor, Tourette's syndrome, stroke (motor, aphasia, hemispatial neglect), amyotrophic lateral sclerosis, multiple sclerosis, epilepsy, Alzheimer's disease, tinnitus, and widespread use in psychiatry, as stated by Lefaucheur et al. [29].

### Oral cavity disorders and brain stimulation applications

Oral health status reflects the functional equilibrium of the muscular tissues, bone, tooth, and mucosa. The most prevalent impaired function in the elderly, after physiological aging, is mastication, which is primarily brought on by tooth loss [30]. The quality of life is reduced if other functions, such as eating, speaking, tasting, and swallowing, are also affected. Decreased motor control, fragile oral tissues, and reduced neuroplasticity are physiological signs of aging in the head and neck area [30]. Moreover, chronic diseases and systemic drugs frequently present in elderly persons also impact the oral cavity and functions. An oral mucosa disease with a higher prevalence in ageing patients is burning mouth syndrome (BMS), considered a chronic nociplastic orofacial pain [31]. The management of BMS does not involve standardized treatment protocols. Instead, a combination of pharmacological therapies, such as topical benzodiazepines, systemic psychotropic agents, and antioxidants, along with supportive methods such as behavioral and psychotherapy, photobiomodulation, acupuncture, and brain stimulation, are used [32].

A randomized controlled single-blind study using rTMS reported a significant pain decrease after 2 weeks of treatment compared to sham stimulation [33]. However, there have been inaccuracies and small errors in studies using TMS and tDCS, and there are limited controlled clinical investigations with healthy participants. In a meta-analysis of five trials that included 270 participants, there was no significant difference in pain severity between active stimulation and sham stimulation (an inactive type of stimulation intended to create the impression of normal stimulation without visible effects): the standard mean difference (SMD) was 0.24 with a 95% confidence interval of -0.48 to 0.01. One research comparing tDCS with sham on 36 people found a good outcome on short-term quality of life (SMD -25.05, 95% CI - 37.82 to -12.28, very poor quality). Another study found that tDCS decreased post-stroke pain. Compared to tDCS alone, a single session decreased neurogenic arm pain by over two-thirds (36.5% *vs*. 15.5%) [34].

Numerous studies suggest that using tDCS to treat neuropsychiatric disorders affects sleep neurophysiology. Due to the wide variety of stimulation regimens for neuropsychiatric disorders, it is essential for future research to investigate the analgesic, sleep-enhancing, and cognitive-enhancing effects of tDCS in healthy adults. In animal experiments, new protocols such as theta burst stimulation (TBS) are used to induce synaptic plasticity. TBS is based on the hippocampus' inherent theta rhythm [35] and involves short episodes of high stimulation ("bursts of stimulation"). Boost doses of 80% to 90% of the resting motor threshold (RMT) are common; however, process variables and interindividual differences must also be considered. Depending on the desired effect, well-defined TMS sites are stimulated. Chronic pain studies use the main motor cortex (M1). When activated, it causes the motor homunculus's muscle groups to contract, making it easy to verify activation. The resting motor threshold (RMT) is the minimum stimulation intensity required to elicit a motor-evoked potential in 50% of calm individuals. The dorsolateral prefrontal cortex (DLPFC) is a commonly targeted brain region in behavioral and psychiatric studies, especially in depression. Moreover, it has also been investigated in studies related to fibromyalgia. Additional areas stimulated include the premotor cortex, the supplementary motor area for Parkinson's patients, the prefrontal cortex for schizophrenia or Parkinson's disease, the posterior parietal cortex for hemispatial neglect, the temporal cortex for tinnitus, and the temporoparietal cortex for tinnitus or auditory hallucinations [36].

### Case study – the use of TMS and neuromodulation in the therapy of chronic pain syndrome in glossodynia

There are few and small studies on the role of TMS and other non-invasive neuromodulation methods in glossodynia pain therapy.

In the first study carried out in 2015, a number of 20 patients diagnosed with glossodynia were randomized into two groups (12 subjects and 8 subjects). The first group underwent a left DLPFC stimulation rTMS protocol in which they received 30,000 pulses at a frequency of 10 Hz (15-minute sessions/day, 3000 pulses/day, for 10 days). The pain syndrome, the functionality of the patients, and the affective state were evaluated for 2 months after the end of the stimulation sessions. The second group of 8 patients underwent a sham protocol [37]. According to the authors, there was a significant decrease in pain sensation (on average, 67% and 75% of patients reported a decrease in pain intensity measured on the visual analogue scale (VAS) by more than half compared to before the procedure). The most significant decrease in pain sensation occurred in the first week after stimulation. The degree of functionality of the patients in the first group had a similar tendency, correlating with the intensity of the decrease in symptoms. No significant or serious side effects were reported. In the case of the control group, pain complaints remained unchanged [37].

As part of this research, a case study was also documented of a 64-year-old patient with manifestations of glossodynia for almost a decade. The patient was investigated extensively without identifying an oral pathology or major organic suffering to explain the symptomatology. The recommended drug therapy included, over time, antibiotics, topical dexamethasone, opioids, several generations of antidepressants (tricyclics, SSRIs, SNRIs), and anxiolytics, but without success. Prior to performing magnetic stimulation therapy, the patient underwent a complete medical evaluation, with no cause (oral, organic, or psychiatric) identified for the persistent glossodynia. Pain sensations fluctuated during therapy, but towards the end, there was a robust effect of reducing the pain sensations, the tendency being maintained in the following two months after the end of therapy. However, xerostomia and migraines accompanying glossodynia were not influenced by rTMS [38].

TMS appears to be a promising therapy for patients with resistant glossodynia. It has few side effects, and it is generally well tolerated. In addition, TMS can be used in conjunction with classic pharmacological therapies on an outpatient basis, and it is relatively inexpensive, especially if we report it during the period of manifestation of the therapeutic effect [39].

Despite all these benefits, the method is still far from becoming a standard in the therapy of burning mouth syndrome, especially due to the absence of validated therapeutic protocols, the relatively low spread of the method, the limited access to equipment and software programs, and, above all, inadequate training and experience of medical staff. The high variability of results obtained in non-invasive stimulation sessions can be mitigated by laborious stratification of patients and additional assessments, but even under these conditions, there is a significant dispersion of the therapeutic effect. Furthermore, some patients may experience vegetative reactions during the initial sessions, leading to reluctance and later abandonment of the therapy [39].

## CONSIDERATIONS REGARDING THE USE OF MUSCLE COMPUTER INTERFACES (MUCIS) AND NON-INVASIVE TRANSCRANIAL MAGNETIC STIMULATION

A muscle-computer interface, or muCI for short, is a human-machine system that allows a computer to connect with a user via the use of electromyographic impulses. Signals derived from surface electromyography (sEMG) are being used to give commands to robotic devices such as robotic arms and hands, as well as mobility robots such as wheelchairs. In our case, the subject's health before, during, and after treatment might be monitored utilizing muscle-computer interfaces (muCIs) at the masticatory and temporal muscles to redesign or enhance therapy sessions and identify when therapeutic operations should cease. MuCI human-machine systems use EMG signals to allow humans to operate robotic equipment [37], whereas the suggested application might record human muscle responses in EMG-based active treatments to acquire a thorough knowledge of the subject's neurophysiology and clinical picture.

Real-time computer feedback, utilizing clinical exam data and medical records, has the potential to enhance the precision of TMS and tDCS treatments. Artificial intelligence models can analyze this data to identify patterns and make recommendations that can be used to optimize future treatment plans and stimulation patterns. Motor control could be improved via muCis using muscle biofeedback to train subjects to avoid unwanted simultaneous activation of antagonist muscles (co-contractions), a procedure that can also be used in medical recovery or training of athletes.

"Human in the loop" is a term used to describe the process of having humans review AI output, such as experts, astronauts, and others. This approach helps prevent machine errors and adjust low-confidence forecasts. Fine-tuning the simulation profile is essential to achieving therapeutic effects with TMS and tDCS. There are several methods available to test mastication muscles, and surface electromyography (sEMG) is a commonly used approach in muscle-computer interfaces (muCIs) for at-home telerehabilitation and to operate robotic equipment, such as wheelchairs [40, 41].

Electromyography, often known as EMG, is a technique that measures the aggregate electric signal from muscles. These signals are created during muscular contraction and are regulated by the neurological system. The signal is a representation of the anatomical and physiological features of the muscles, and the EMG signal is the electrical activity of the motor units inside a muscle. There are two types of EMG signals: surface EMG (sEMG) and intramuscular EMG (iEMG). sEMG measures masticatory muscle response to investigate masticatory and temporal neuromotor features. EMG activation of the masseter and anterior temporal muscles is unaffected by resting posture or bilateral mastication [42].

sEMG is a diagnostic tool that offers continual and reliable muscle activity evaluation and may be used to diagnose temporomandibular disorders, a subtype of orofacial pain disorders. Surface-detected signals may be recorded by invasive electrodes as well as non-invasive electrodes, and they are the preferred method for acquiring information on the duration or magnitude of activation of superficial muscle layers.

Sonomyography is an ultrasonic imaging method lately used as an alternative to sEMG to sense muscle activation [43, 44]. Sarcopenia is associated with masseter and temporalis muscle thickness [45, 46]. Because sonomyography (or ultrasonic sensing) depends on the mechanical deformation of muscles to regulate position, we think this control mechanism is compatible with proprioceptive input from muscles. The biting force may be measured using literature and commercially available instruments. This apparatus can measure the maximal voluntary occlusal bite force (MVOBF) [47, 48].

Intraoral scanning (IOS) [49] is a digital orthodontic therapy that reduces process time, which may boost clinical effectiveness and patient comfort. IOS reduces working time, improves patient-reported results, and provides precise digital castings, reducing the possibility of deformation from impression materials. Combining IOS data with CAD and CAM creates a digital workflow.

To determine the optimal amount of automation and human involvement in the System of Interest (SoI), it is crucial to consider the perspective of the "person in the loop". Human Dependability (HUDEP) concepts and methods [50] can be used to guide this decision-making process. While an efficient and automated SoI can improve process efficiency, there is a trade-off between automation and human involvement. Too much automation may lead to a loss of situational awareness for the operator, while inadequate automation can exclude a significant amount of data. Prioritizing the human component in loop-based systems can reduce mistakes caused by process automation and allow for intervention when necessary [51].

## Conclusions

Physiological changes that occur in organisms after prolonged exposure to microgravity are challenging to reproduce under similar conditions on Earth. Identifying similarities between physiological and pathophysiological processes observed in humans or animals during spaceflight and those encountered in research and medical settings is a more cost-effective approach. Our proposed research aims to develop a system that can evaluate different aspects of oral diseases associated with pain. The finding of masticatory muscle properties that have resistance to sarcopenia in contrast to other skeletal muscle groups is the first field of investigation that should be considered. In the context of spaceflight, researchers have explored the development of therapeutic protocols for pain management in the orofacial region using non-invasive transcranial magnetic stimulation as a non-pharmacological alternative. The methods of TMS and tDCS will be applied to oral pathology and biomechanics, with a human participant incorporated into the feedback loop. The objective is to establish therapeutic TMS/tDCS models based on functional, neurophysiological, and clinical evaluation procedures, with human feedback, to enhance their level of confidence. This will be accomplished by combining these three types of evaluation procedures.
